# Intestinal *α-glucosidase* and some pancreatic enzymes inhibitory effect of hydroalcholic extract of *Moringa stenopetala* leaves

**DOI:** 10.1186/1472-6882-14-180

**Published:** 2014-06-03

**Authors:** Alemayehu Toma, Eyasu Makonnen, Yelamtsehay Mekonnen, Asfaw Debella, Sirichai Addisakwattana

**Affiliations:** 1 Pharmacology Department, School of Medicine, Addis Ababa University, Addis Ababa, Ethiopia; 2 Pharmacology Unit, School of Medicine, Hawassa University, P O Box 1560, Hawassa, Ethiopia; 3 Department of Biology, College of Natural Sciences, Addis Ababa University, Addis Ababa, Ethiopia; 4 Department of Traditional and Modern Drug Research, Ethiopian Public Health Institute (EHPI), Addis Ababa, Ethiopia; 5 The Medical Food Research and Development Center, Department of Nutrition and Dietetics, Faculty of Allied Health Sciences, Chulalongkorn University, Pathumwan 10330 Bangkok, Thailand

**Keywords:** *Moringa stenopetala*, Phytochemical analysis, α-glucosidase, Pancreatic enzymes

## Abstract

**Background:**

*Moringa stenopetala* has been used in traditional health systems to treat diabetes mellitus. One of the successful methods to prevent of the onset of diabetes is to control postprandial hyperglycemia by the inhibition of α-glucosidase and pancreatic α-amylase activities, resulting in the aggressive delay of the carbohydrate digestion of absorbable monosaccharides. The aim of the present study is to investigate the effect of the extract of the leaves of *Moringa stenopetala* on α-glucosidase, pancreatic α-amylase, pancreatic lipase, and pancreatic cholesterol esterase activities, and, therefore find out the relevance of the plant in controlling blood sugar and lipid levels.

**Methods:**

The dried leaves of Moringa stenopetala were extracted with hydroalcoholic solvent and dried using rotary vapor under reduced pressure. The dried extracts were determined for the total phenolic compounds, flavonoid content and condensed tannins content by using Folin-Ciocateu’s reagent, AlCl_3_ and vanillin assay, respectively. The dried extract of plant-based food was further quantified with respect to intestinal α-glucosidase (maltase and sucrase) inhibition and pancreatic α-amylase inhibition by glucose oxidase method and dinitrosalicylic (DNS) reagent, respectively.

**Results:**

The phytochemical analysis indicated that flavonoid, total phenolic, and condensed tannin contents in the extract were 71.73 ± 2.48 mg quercetin equivalent/g of crude extract, 79.81 ± 2.85 mg of gallic acid equivalent/g of crude extract, 8.82 ± 0.77 mg catechin equivalent/g of crude extract, respectively. The extract inhibited intestinal sucrase more than intestinal maltase with IC_50_ value of 1.47 ± 0.19 mg/ml. It also slightly inhibited pancreatic α-amylase, pancreatic lipase and pancreatic cholesterol esterase.

**Conclusion:**

The result demonstrated the beneficial biochemical effects of *Moringa stenopetala* by inhibiting intestinal α-glucosidase, pancreatic cholesterol esterase and pancreatic lipase activities. A daily supplement intake of the leaves of Moringa stenopetala may help in reducing hyperglycemia and hyperlipidemia.

## Background

Worldwide, the number of people with diabetes and pre-diabetes is exponentially increasing mainly due to aging, urbanization, unhealthy eating habits, increasing prevalence of obesity and lack of physical activity [1]. Diabetes mellitus is a leading cause of morbidity and mortality worldwide, with an estimated 382 million adults being affected and 5.1 million people killed in the year 2013. The prevalence is expected to be 592 million in the year 2035, with the greatest increases expected in low- and middle-income developing countries of the African, Asian, and South American regions. At present, 80% of the worlds’ populations with diabetes live in low- and middle income countries [2]. Diabetes is also associated with a host of life threatening and potentially disabling macro- and micro-vascular complications [3]. Hence, there is a much larger burden in the form of loss of productivity as a result of restricted daily activity which results in high economic costs.

*Moringa stenopetala (Baker f) Cufodontis* belongs to family Moringaceaeis commonly grown in Southern parts of Ethiopia [4]. The leaves of Moringa stenopetala are cooked and eaten as vegetables and the leaves and roots are used to treat malaria, diabetes, asthma, repelled placenta, hypertension and gastrointestinal problems [5,6]. It has been reported that *Moringa stenopetala* leaves and roots showed antitrypanosomal activity [7]. The antispasmodic effects of the leaves on smooth muscle tissues and antibiotic properties of the seeds [5,8] have also been reported. The crude aqueous extract of the leaves demonstrated hypoglycemic activity [9]. The crude aqueous/ethanol extract and fractions of the leaves of *Moringa stenopetala* have been reported to have both hypoglycemic and antihyperglycemic effect [10,11]. Moreover, chronic administration of the n-butanol fraction of ethanol extract of *Moringa stenopetala* leaves in alloxan-induced diabetic mice showed antihyperglycemic and antihyperlipedimic effects with wide margins of safety, indicating its potential for long term management of diabetes [12].

The aim of the present study was to investigate the inhibitory effect of the leaf extract of *Moringa stenopetala* on α-glucosidase, pancreatic α-amylase, pancreatic lipase, and pancreatic cholesterol esterase activities besides phytochemical analysis.

## Methods

### Chemicals

Folin-Ciocalteu, quercetin, catechin, gallic acid, rat intestinal acetone powder, porcine pancreatic α-amylase, vanillin, 3,5-dinitrosalicylic acid, glucose oxidase kits, p-nitrophenylbutylrate (p-NPB), p-nitrophenylpalmitate (p-NPP), taurodeoxycholic acid, taurocholic acid, porcine cholesterol esterase, porcine pancreatic lipase were purchased from Sigma-Aldrich Co. (St. Louis, MO, USA). All others chemicals used were of analytical grade.

### Collections and preparation of plant materials

The leaves of *Moringa stenopetala* was collected from Gamo Gofa Zone, South Nation’s Nationalities Peoples Region, 520 kilometer south of Addis Ababa. After collection, the plant was identified and authenticated by a taxonomist, and deposited in herbarium of Ethiopian nutrition and health research institute (ENHRI) with a voucher number AL-001. It was then dried under shade and crushed to powder for extraction.

### Preparation of plant material extract

The powdered leaves (1.2 Kg) were extracted by percolation using 70%( v/v) ethanol, and the mixture was then filtered using Whatmann filter paper no. 1. The extract was dried by evaporation using rotary vaporizers under reduced pressure at a temperature of 40-45°C. The residue filtrate obtained was then dried by steam bath at 40°C and kept in refrigerator at 8°C for experimental usage. The yield of the extract was 20.1% in weight by weight (w/w).

### Determination of flavonoid content

Estimation of flavonoid content in the dried extracts was done according to a previous method [13]. The dried extract (0.5 mg) was dissolved in 80% ethanol (1 ml). The sample solution (50 μl) was added to 10 μl of AlCl_3_ solution (10% w/v) and 10 μl of 1 M sodium acetate in absolute ethanol (150 μl). After incubation at 30°C for 30 min, the absorbance was measured immediately at 430 nm. The estimation of flavonoid content was calculated from a calibration curve using quercetin as a standard. The results were expressed as milligram quercetin equivalent/gram dry weight of extract.

$$\mathrm{Flavonoid}\left(\mathrm{mg}/g\right)=\frac{\left(\mathrm{Abs}\;\mathrm{test}\;\mathrm{sample}‒\mathrm{Abs}\;\mathrm{blank}\right)‒\mathrm{Intercept}}{\mathrm{Slope}\times \;\mathrm{amount}\;\mathrm{of}\;\mathrm{sample}\;\mathrm{in}\;\mathrm{gram}}$$

Where Abs test sample was the absorbance of extract with reagent, Abs blank was the absorbance of extract without reagent.

### Determination of total phenolic content

Total phenolic content of the extract was performed according to a previous method [13]. The dried extract (0.5 mg) was dissolved in distilled water (1 ml). The sample solution (50 μl) was mixed with 50 μl of Folin-Ciocateu’s reagent followed by 50 μl of Na_2_CO_3_ (10% w/v). After incubation at 30°C for 60 min, the absorbance was measured at 760 nm using a microplate reader. Total phenolic content was calculated from a calibration curve using gallic acid as a standard. The results were expressed as milligram gallic acid equivalent/gram dry weight of extract.

$$\begin{array}{l} \mathrm{Total}\;\mathrm{phenolic}\;\mathrm{compounds}\left(\mathrm{mg}/g\right)\\ \;=\frac{\left(\mathrm{Abs}\;\mathrm{test}\;\mathrm{sample}‒\mathrm{Abs}\;\mathrm{blank}\right)‒\mathrm{Intercept}}{\mathrm{Slope}\times \mathrm{amount}\;\mathrm{of}\;\mathrm{sample}\;\mathrm{in}\;\mathrm{gram}} \end{array}$$

Where Abs test sample was the absorbance of extract with reagent, Abs blank was the absorbance of extract without reagent.

### Determination of condensed tannin content

Estimation of condensed tannin content in the dried extracts was done according to a previous method [14]. The dried extract (5 mg) was dissolved in 80% ethanol (1 ml). The sample solution (50 μl) was added to 100 μl of vanillic acid solution (4% w/v) and 50 μl of concentrated HCl. The absorbance was measured immediately at 500 nm. The estimation of condensed tannin content was calculated from a calibration curve using catechin as a standard. The results were expressed as milligram catechin equivalent/gram dry weight of extract.

$$\mathrm{Condensed}\;\mathrm{tannins}\left(\mathrm{mg}/g\right)=\frac{\left(\mathrm{Abs}\;\mathrm{test}\;\mathrm{sample}‒\mathrm{Abs}\;\mathrm{blank}\right)‒\mathrm{Intercept}}{\mathrm{Slope}\times \mathrm{amount}\;\mathrm{of}\;\mathrm{sample}\;\mathrm{in}\;\mathrm{gram}}$$

Where Abs test sample was the absorbance of extract with reagent, Abs blank was the absorbance of extract without reagent.

### Pancreatic α-amylase inhibitory activity

The pancreatic α-amylase inhibition assay was performed according to a previous report [15]. Porcine pancreatic α-amylase (3units/ml) was dissolved in 0.1 M phosphate buffer saline, pH 6.9. The various concentrations of the extract (10 μl) were added to a solution containing starch (1 g/l) and phosphate buffer (165 μl). The reaction was initiated by adding enzyme solution (75 μl) to the incubation medium. After 10 min incubation, the reaction was stopped by adding 250 ml dinitrosalicylic (DNS) reagent (1% 3, 5-dinitrosalicylic acid, 0.2% phenol, 0.05% Na_2_SO_3_ and 1% NaOH in aqueous solution) to the reaction mixture. The mixtures were heated at 100°C for 10 min in order to stop the reaction. Thereafter, 250 μl of 40% potassium sodium tartarate solution was added to the mixtures to stabilize the color. After cooling to room temperature in a cold water bath, the absorbance was recorded at 540 nm using a microplate reader. Acrabose was used as positive control.

$$\%\mathrm{Inhibition}=\frac{\mathrm{AbsContol}‒\mathrm{AbsSample}\times 100}{\mathrm{AbsControl}}$$

Where Abs Control was the absorbance without sample, Abs samples was the absorbance of sample extract.

### Intestinal α-glucosidase inhibitory activity

The assessment of intestinal α-glucosidase inhibitory activity was based on the modified method previously described [15]. Briefly, 100 mg of rat intestinal acetone powder was homogenized in 3 ml of 0.9% NaCl solution. The solution was centrifuged at 12,000 g for 30 min and then subjected to assay. The crude enzyme solution (as maltase assay, 10 μl; as sucrase assay, 30 μl) was incubated with 30 μl maltose (86 mM) or 40 μl sucrose (400 mM), 10 μl of the extract at various concentrations, followed by the addition of 0.1 M phosphate buffer, pH 6.9 to give a final volume of 100 μl. The reaction was incubated at 37°C for 30 min (maltase assay) or 60 min (Sucrase assay). Thereafter, the mixtures were suspended in boiling water for 10 min to stop the reaction. The concentrations of glucose released from the reaction mixtures was determined by glucose oxidase method with absorbance at a wavelength of 450 nm. Intestinalα-glucosidase inhibitory activity was expressed as percentage inhibition using the following formula. Acrabose was used as positive control.

$$\%\mathrm{Inhibition}=\frac{\mathrm{AbsControl}‒\mathrm{AbsSample}}{\mathrm{AbsControl}}\times 100$$

Where Abs Control was the absorbance without sample, Abs samples was the absorbance of sample extract.

### Pancreatic cholesterol esterase inhibition

The pancreatic cholesterol esterase inhibition was performed spectrophotometrically based on previous method [13]. The extract was incubated with mixtures containing 5.16 mM taurocholic acid, 0.2 mM p-NPB in 100 mM sodium phosphate buffer, 100 mM NaCl, pH 7.0. The reaction was initiated by adding porcine pancreatic cholesterol esterase (1 μg/ml). After incubation for 5 min at 25°C, the mixtures were measured the absorbance at 405 nm. Simvastatin was used as positive control.

$$\%\mathrm{Inhibition}=\frac{\mathrm{AbsControl}‒\mathrm{AbsSample}}{\mathrm{AbsControl}}\times 100$$

Where Abs Control was the absorbance without sample, Abs samples was the absorbance of sample extract.

### Pancreatic lipase inhibition

The pancreatic lipase inhibition was performed spectrophotometrically based on previous method with little modification [16]. The extract was incubated with mixtures containing 5 mM deoxytaurocholic acid, 0.2 mM p-NPP in 50 mM sodium phosphate monobasic buffer, pH 8.0. The reaction was initiated by adding porcine pancreatic lipase (10 mg/ml). After incubation for 5 min at 37°C, the mixtures were measured the absorbance at 410 nm. Orlistat was used as positive control.

$$\%\mathrm{Inhibition}=\frac{\mathrm{AbsControl}‒\mathrm{AbsSample}}{\mathrm{AbsControl}}\times 100$$

Where Abs Control was the absorbance without sample, Abs samples was the absorbance of sample extract.

### Data analysis

The IC50 values were calculated from plots of log concentration of inhibitor concentration versus percentage inhibition curves using sigma plot version11. The data were expressed as mean ± standard error of mean (SEM).

## Results

The amount of total flavonoids, total polyphenolic compounds, and condensed tannins contents determined in the extract are summarized in Table 1. The content of flavonoid in the extract was 71.73 ± 2.48 mg quercetin equivalent/g of crude extract. The total polyphenolic compound in the extract was 79.81 ± 2.85 mg of gallic acid equivalent/g of crude extract. Furthermore, the content of condensed tannins in the extract was found to be 8.82 ± 0.77 mg catechin equivalent/g of crude extract.

**Table 1:** Flavonoid, total phenolic, and condensed tannin contents of Moringa stenopetala leaves extract

**Phytochemical analysis**			
	**Flavonoid (mg/g extract)**	**Total phenolics (mg/g extract)**	**Condensed tannins (mg/g extract)**
***Moringa stenopetala***	71.73 ± 2.48	79.81 ± 2.85	8.82 ± 0.77

Results are expressed as means ± S.E.M., n = 3.

As shown in Table 2, the IC50 value of the extract was 1.47 ± 0.19 mg/ml for intestinal sucrase, where as the extract (5 mg/ml) inhibited intestinal maltase by 48.64 ± 1.18%. The findings indicated that the extract was a more specific inhibitor of intestinal sucrase than intestinal maltase. The extract also inhibited pancreatic cholesterol esterase activity (49.22 ± 2.34) more than pancreatic α-amylase (6.06 ± 0.75%). Moreover, it was found that the extract inhibited pancreatic lipase with IC50 value of greater than 5 mg/ml.

**Table 2:** The inhibitory effects of Moringa stenopetala leaf extract on pancreatic α-amylase, maltase, sucrase, pancreatic lipase, and pancreatic cholesterol esterase activities

**IC**_**50 **_**values (mg/ml)**					
	**Pancreatic α-Amylase**	**Maltase**	**Sucrase**	**Pancreatic lipase**	**Pancreatic cholesterol esterase**
***Moringa stenopetala***	> 5	> 5	1.47 ± 0.19	>5	>5

Results are expressed as means ± S.E.M., n = 3.

## Discussion

Diabetes mellitus is a metabolic disorder that usually affects carbohydrate, fat, and protein metabolism, followed by multiorgan injury in the later period, and hyperipidemia is associated with hyperglycemia [17]. More powerful new compounds with pan-target activity and proven long-term safety should be highly effective in a clinical setting for patients with coexisting relevant lipid and glucose metabolic disorders. These discoveries pave the way for the development of drugs for treating chronic multigenic metabolic and cardiovascular diseases, for which therapy is presently insufficient or nonexistent [18]. This is the first study to investigate the effect of leaf extract of *Moringa stenopetala* on pancreatic and intestinal enzymes related to antihyperglycemic and antihyperlipidemic actvities.

The presence of phytochemicals in plant products gives a great potential for balancing metabolic disturbances. Several phytomolecules including flavonoids, phenolic compounds, alkaloids, glycosides, saponins, glycolipids, dietary fibres, polysaccharides, peptidoglycans, carbohydrates, amino acids and others obtained from various plant sources have been reported as potent hypoglycemic agents. Flavonoids are a heterogeneous group of ubiquitous plant polyphenols, which exhibit a variety of pharmacological activities, including the anti-atherogenic as well as antihyperglycemic effects, lipoprotein oxidation, blood platelet aggregation and vascular reactivity [19]. A high content of phytochemicals especially total polyphenolic compounds and total flavonoids may contribute to the pleiotropic effects of *Moringa stenopetala* leaves that support the use of the plant for different metabolic disorders in the local community.

It is well known that inhibition of intestinal α-glucosidase and pancreatic α-amylase activity results in delaying carbohydrate digestion of absorbable monosaccharides, causing reduction of postprandial hyperglycemia. The plant extract showed a weaker pancreatic α-amylase activity compared to intestinal α-glucosidase activity. α-glucosidase inhibitors delay intestinal carbohydrate absorption and slow the sharp rise in blood sugar levels that diabetic patients typically experience after snacks. However, none of the currently available α-glucosidase inhibitors for clinical use are devoid of severe adverse effects [20,21]. The search for new group of agents from natural resources especially from traditional medicines has, therefore, become an attractive approach for the treatment of postprandial hyperglycemia. Our study showed that hydroalcoholic leaf extract of Moringa stenopetala is a potent inhibitor of α-glucosidase activity, and therefore suggests that extracts of *Moringa stenopetala* could be an attractive source of alternative treatment.

Besides hyperglycemia, diabetes mellitus is highly characterized by elevated levels of triglycerides and cholesterol in the blood highly associated with a modern lifestyle and increase consumption of a high fat diet [22]. Reducing absorption of free fatty acids and free cholesterol by inhibiting pancreatic lipase and pancreatic cholesterol esterase reduces hyperlipidemia associated with diabetes mellitus [23,24]. In our previous findings it was reported that the plant material of *Moringa stenopetala* has antihyperlipidemic effects [12] which may be due to the inhibition of pancreatic lipase and pancreatic cholesterol esterase.

## Conclusion

In conclusion, we demonstrate here that the inhibition of intestinal α-glucosidase by extracts of *Moringa stenopetala* may contribute to antihyperglycemic activity. Extracts of *Moringa stenopetala* also show antihyperlipidemic activity due to the inhibition of lipase and cholesterol esterase enzymes. Thus plant material of *Moringa stenopetala* could be used for prevention/treatment of hyperglycemia and hyperlipidemia. Further illustration on mechanism(s) of *Moringa stenopetala* leaves on insulin secretion and plasma lipid inhibition on animal models, and role of the plant material for management of protein glycation are being investigated.

## Competing interests

The authors declare that they have no competing interests.

## Authors’ contribution

AT conceived the idea, drafted the proposal and involved in all implementation stages of the project and write up. EM, YM, AD and SA reviewed the proposal, and involved in all implementation stages of the project and write up. All authors reviewed the proposal and the final manuscript. All authors approved final version of the manuscript.

## Pre-publication history

The pre-publication history for this paper can be accessed here:

http://www.biomedcentral.com/1472-6882/14/180/prepub
